# Supplementary figures and images for: The Effect of Alternative Splicing Sites on Mirtron Formation and Arm Selection of Precursor microRNAs
Source: Int J Mol Sci. 2024 Jul 12;25(14):7643. doi: 10.3390/ijms25147643 (PMC11277307; doi:10.3390/ijms25147643)

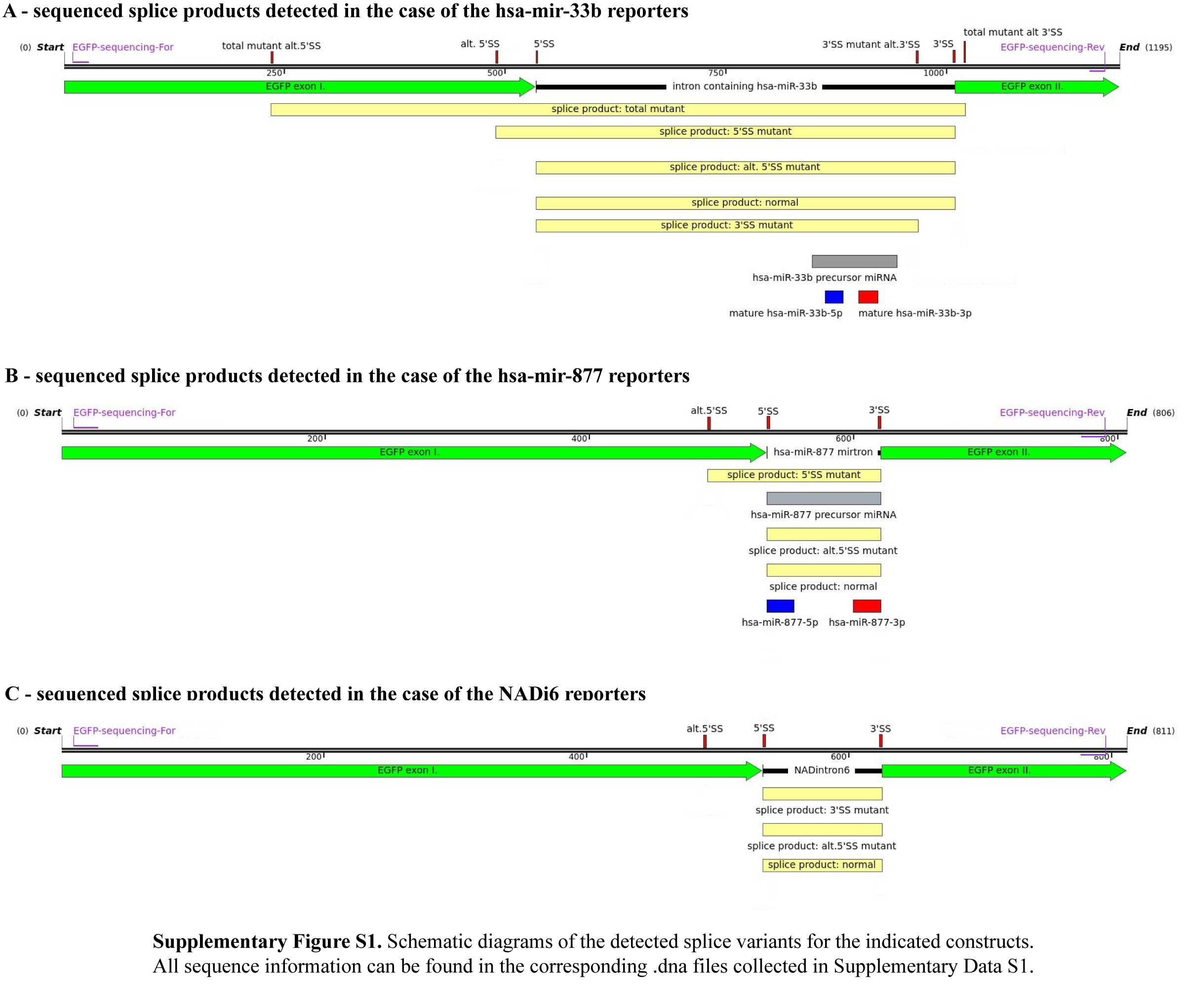

Supplement: Supplementary file 1 [file ijms-25-07643-s001.zip › Suppl_Figure_S1.tif]

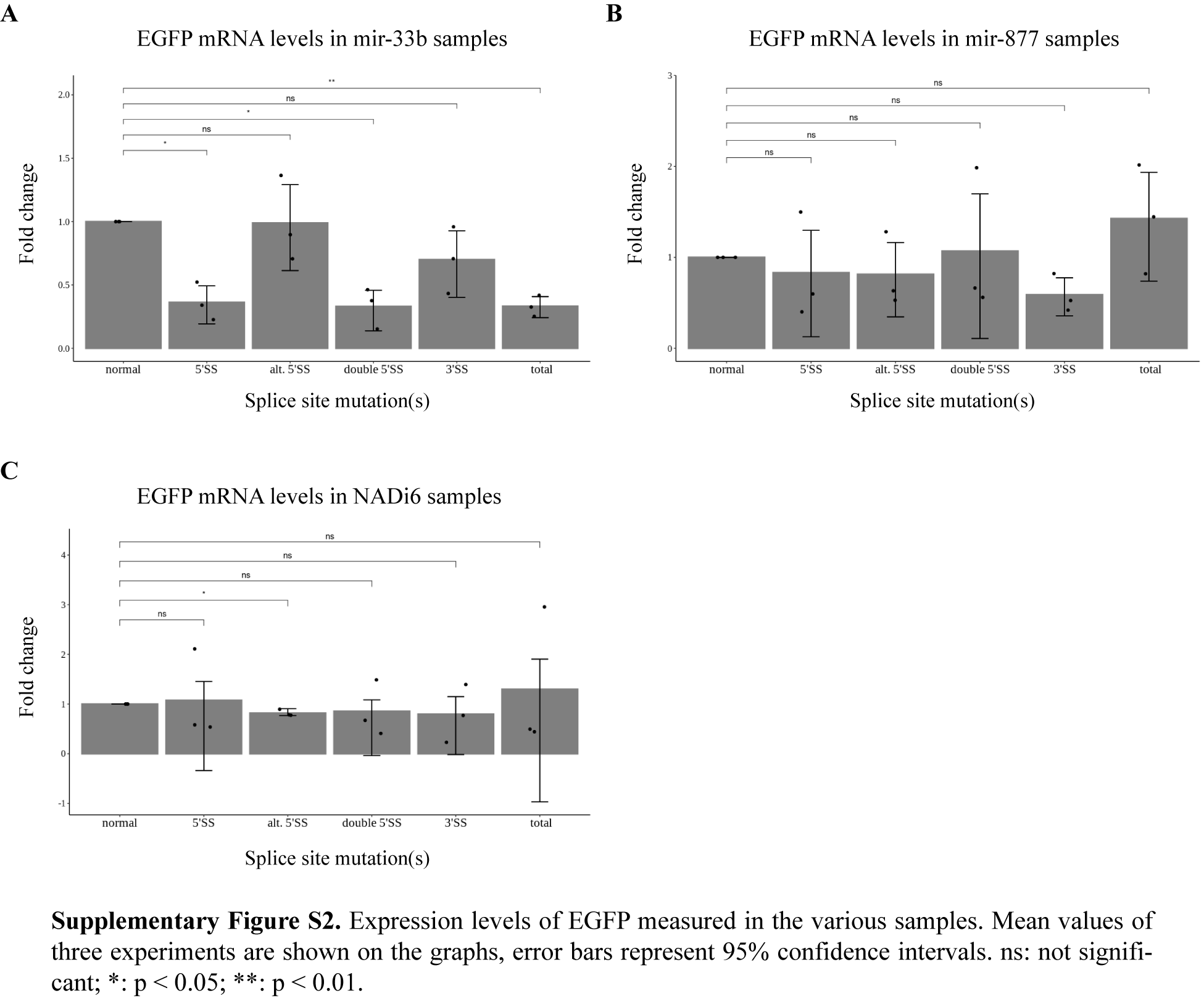

Supplement: Supplementary file 1 [file ijms-25-07643-s001.zip › Suppl_Figure_S2.tif]
